# Voice Technologies to Support Aging in Place: Opportunities and Challenges

**DOI:** 10.1093/geroni/igaa057.1016

**Published:** 2020-12-16

**Authors:** Alisha Pradhan, Amanda Lazar

**Affiliations:** 1 University of Maryland, College Park, College Park, Maryland, United States; 2 University of Maryland, College Park, Maryland, United States

## Abstract

Technology to support aging in place has been a topic of interest. Research indicates that older adults are increasingly using commercially available voice assistants in smart speakers. These devices enable non-visual interaction that does not require extensive expertise with traditional mobile or desktop computers, thus offering new possibilities of access to digital technology. We conducted two different studies with individuals aged 65 years old or above—a three week smart speaker deployment study with individuals who did not use computing devices regularly and a workshop on customizing internet of things technology with tech savvy individuals. Our findings indicate specific ways that these voice technologies might support aging in place, including ease of use and due to their not being identified with aging-specific technologies. We observed that participants consistently used their voice agent for finding online information, particularly health-related, emphasizing the need to revisit concerns about credibility of information with this new interaction medium. And, although features to support memory (e.g., setting timers, reminders) were initially perceived as useful, the actual usage was unexpectedly low due to reliability concerns. Our work provides a basis to understand older adults’ perceptions and usage of current voice technologies. We also identify opportunities for customizing voice technologies to better support aging in place.

